# The National Pension Fund

**Published:** 1887-11-12

**Authors:** 


### THE NATIONAL PENSION FUND.

### THE REGISTRATION OF NAMES.

It will save a good deal of trouble if nurses and other hospital officials who propose joining the National Pension Fund will send their names and other particulars to the *Secretary of The Hospitals Association*, Norfolk House, Norfolk Street, W.C., and not to the Editor of The Hospital. Names continue to arrive in considerable numbers, but if those interested desire the Fund to be started before the end of the Jubilee year they must influence and interest all their friends immediately.

### AN AFTERNOON CONFERENCE.

To meet the wishes of many interested in this Fund who reside away from London it has been determined to hold an afternoon conference during the first fortnight in December on a day yet to be fixed. Meanwhile, any who wish points to be cleared up, or who have found a difficulty in understanding the scheme, will facilitate its successful initiation by communicating them in writing to *The Secretary*, as above.

### OPINIONS OF THE PRESS.

### Daily News.

The great meeting in the Society of Arts last week to promote the scheme for founding a National Pension Fund for Nurses and Hospital Officials is believed to have been the largest gathering ever seen in London in connection with hospital work, not excepting the meetings which have been held at the Mansion House. The mere fact that 140 hospitals and nursing institutions were represented, and that 1,200 officials and nurses have already expressed a desire to enrol themselves as members sufficiently testify to the need felt for an organisation of the kind. It is estimated that there are some 15,000 nurses in this country, of whom from one-third to one-half are certificated or hospital-trained. It is obvious that an annual salary of £25, less the cost of washing and some incidental expenses, does not leave much margin for making provision for the future. Nevertheless it is intended that the institution shall partake, to a certain extent, of the character of a provident association. As the hospital nurses give the very prime of their lives to the service of suffering humanity, for wages which would tempt few who are not sustained by the noblest motives, the movement seems eminently worthy of public sympathy and support.

### The Chronicle.

We do not anticipate that any difficulty will be experienced in giving the name of action to the scheme for providing a pension fund for hospital officials and nurses. . . . The causes which have operated to the detriment of the financial position of our hospitals are not likely to prejudice the movement which has been set on foot for providing for hospital officials and nurses when themselves incapacitated for work by sickness or old age. It is the aim of the promoters of the scheme to make it self-supporting by conducting it on the annuity insurance principle. It will be necessary, however, to secure a guarantee fund in order to successfully carry out the proposal.

### The Globe.

Mr. Burdett, so well known in connection with all hospital matters, has put forward a scheme for the benefit of hospital officials which should meet with ready support. It must in the first place be remembered, though the fact is frequently forgotten, that nurses and other hospital officials are as liable to illness as the rest of us. They are constantly doing their best to combat disease and relieve pain in others, but pain and disease sometimes take their revenge. The skilful nurse is herself attacked, in a few short weeks the slender savings which her too scanty pay permits are all exhausted, and she is compelled to depend upon the help of friends, which may not be forthcoming. Then, again, even nurses and attendants are apt to grow old and get past work. To keep them out of the workhouse and provide them with support during illness is the object of the proposed fund. The public, exhausted by the special calls made upon it this year, will be glad to hear that the proposed fund is to be self-supporting. But those, and they are a very large percentage of the population, who have benefited in hospital or at home by the tender ministrations of trained nurses, may also be glad to hear that it will be possible to contribute either to the fund as a whole or to the aid of any particular subscriber to it. The scheme includes a sick fund, a pension fund, and an insurance fund. Its establishment can hardly fail to benefit, not only the nurses themselves, but also the general public, for it will put the whole profession of nursing on a firmer foundation, and render it more attractive to capable women.

### Western Daily Press.

A movement is on foot to found a National Pension Fund for Hospital Officials and Nurses as a provision against sickness and advancing years. The trained nurses are constantly being recruited from the ranks of gentlewomen who have adopted nursing as a career, and many of them find employment at from one to two guineas per week. The occupation of a nurse is very arduous, and the nursing of nurses who have broken down under the strain of heavy work in sick rooms is a question of some importance. Superannuation allowances and pensions are likely to receive much more attention during the next few years than they have hitherto done among all classes of workers, and there is no class to whom such a provision would be more beneficial than the class who have devoted their lives to the relief of the suffering of others.

